# The comparison and validity and reliability study of bilateral innominate vertical length measurements using innovative digital radiographic imaging software in assessing scoliotic leg length discrepancy

**DOI:** 10.1186/1748-7161-9-S1-O9

**Published:** 2014-12-04

**Authors:** Xiaohua He, HanSuk Jung, JooHyun Ham, KyeongAh Min

**Affiliations:** 1 Palmer College of Chiropractic Florida, Port Orange, USA; 2 HanSeo University, Seosan, South Korea

## Background information

The role of leg length discrepancy (LLD) has been implicated in certain types of scoliosis [1]. Scoliotic LLD has been suggested as a result of rotation of the innominate bones [2]. However, the role of innominate vertical lengths (IVLs) as a predisposing factor for scoliosis is not clear. The reliability of radiographic measurements may reveal whether IVL can be used as a factor for clinical assessment.

## Purpose

Clinical investigation of quantifying bilateral IVLs and to assess the intra- and inter-observer variability using digital radiographic techniques.

## Methods

Twenty x-ray films from scoliotic patients with LLD were chosen based on convenience, without predilection for gender and age. Images were examined by 7 trained observers to compare bilateral IVLs and to estimate the variability, as well as intra- and inter-observer variations. Each image was measured 3 times at a minimum interval of 1 week. All radiographs were calibrated by the software to allow for accurate length measurements. Student’s t-test was used to compare bilateral IVLs. The intraclass correlation coefficients (ICC) were used. 95% prediction limits for the errors in measurements to determine the interobserver and intraobserver reliabilities. A mean ICC value of 0.93 was determined for interobserver reliability and a mean ICC value of 0.96 for intraobserver reliability.

## Results

Overall mean right IVL was 192.6 ± 6.94mm, and left IVL was 190.4 ± 6.95mm. Although there was a discrepancy between bilateral IVLs, there was no statistical significance (P>0.05). Interobserver ICC was 0.954 and intraobserver ICC was 0.974.

## Conclusion

Scoliosis patients with LLD might show asymmetrical IVLs; however, this discrepancy has no statistical significance; therefore, IVL is not a strong clinical indicator in assessing scoliotic LLD. On the other hand, the computer-assisted measurements are clinically advantageous and appropriate to assess scoliosis parameters. Digital measurement among different observers showed excellent reliability for the majority of IVL parameters, making it a useful method for the analysis of pathology on radiographs in scoliosis patients.
